# Inhibition of integrin α5β1 ameliorates VEGF-induced retinal neovascularization and leakage by suppressing NLRP3 inflammasome signaling in a mouse model

**DOI:** 10.1007/s00417-018-3940-x

**Published:** 2018-03-03

**Authors:** Ailing Sui, Yisheng Zhong, Anna M. Demetriades, Qing Lu, Yujuan Cai, Yushuo Gao, Yanji Zhu, Xi Shen, Bing Xie

**Affiliations:** 1 The Department of Ophthalmology, Ruijin Hospital, Shanghai Jiao Tong University School of Medicine, Shanghai, China; 2 The Department of Ophthalmology, New York Presbyterian Hospital-Weill Cornell Medicine, New York, USA

**Keywords:** Vascular endothelial growth factor, Integrin α5β1, ATN-161, NLRP3 inflammasome, Retinal neovascularization, Tet/opsin/VEGF transgenic mice

## Abstract

**Purpose:**

To assess the effect of inhibiting integrin α5β1 by ATN-161 on vascular endothelial growth factor (VEGF)-induced neovascularization (NV) and leakage causing retinal detachment in adult Tet/opsin/VEGF transgenic mice, and characterize the underlying mechanism of its function.

**Method:**

Retinas from adult Tet/opsin/VEGF transgenic mice and human retinal endothelial cells (HRECs) exposed to VEGF (treated with ATN-161 or PBS) were used to carry out immunofluorescence, RT-PCR and western blot to examine expression levels of integrin α5β1 and the NACHT, LRR, and PYD domains-containing protein 3 (NLRP3) inflammasome. Retinal frozen section analysis was used to assess NV and leakage causing retinal detachment.

**Results:**

In comparison to normal-treated mice, doxycycline-treated Tet/opsin/VEGF transgenic mice showed severe retinal detachment and higher integrin α5β1 expression. Furthermore, the retinal detachment was inhibited significantly by ATN-161. Additionally, ATN-161 treatment was associated with a conspicuous reduction in NLRP3, apoptosis-associated speck-like protein containing a CARD (ASC), cleaved caspase-1, and mature interleukin-1β expression levels in the retinas of Tet/opsin/VEGF transgenic mice treated with doxycycline as well as in HRECs exposed to VEGF.

**Conclusion:**

ATN-161, an antagonist of integrin α5β1, is a promising treatment for retinal neovascularization (RNV), and its retinal protection role appears to take effect through inhibition of NLRP3 inflammasome activity.

## Introduction

Retinopathy of prematurity (ROP) and diabetic retinopathy (DR) are common retinal neovascular blinding eye diseases [1, 2]. Exudation, bleeding, proliferation, and other pathological changes may occur, along with severe complications such as retinal edema and exudation, vitreous hemorrhage, and retinal detachment, resulting in irreversible visual impairment and even permanent loss of vision [3]. Preventing and treating retinal neovascularization (RNV) remains an enormous challenge. Some studies have provided novel ideas for understanding the complex pathological mechanism of RNV. The most widely accepted viewpoint is that hypoxia increases vascular endothelial growth factor (VEGF) expression and then causes neovascularization (NV). Currently, anti-VEGF therapy remains an effective pharmacotherapy and is beneficial for some ocular NV patients in clinical application. However, anti-VEGF therapy is expensive and drug-related complications such as resistance and nonresponse exist, and its application is also limited [4–7]. Thus, understanding the molecular mechanisms underlying the regulation of RNV is required for the development of safe and effective antiangiogenic therapies. Integrins, a family of enzymatically inactive cell adhesion receptors, consist of 19 different α subunits and 8 different β subunits. Thus far, these subunits form at least 25 different integrins with distinct ligand-binding specificities [8] and play significant roles in both cell-cell and cell-extracellular matrix interactions and modulate various signaling pathways involving cell adhesion, migration, differentiation, and angiogenesis [9, 10]. Integrin α5β1 is a specific receptor of fibronectin [9]. Both integrin α5β1 and fibronectin are upregulated in growth factor-induced NV [11], whereas the expression of integrin α5β1 is low in quiescent vascular cells [11, 12]. Prior research showed that integrin α5β1 inhibition by ATN-161 significantly decreased choroidal neovascularization (CNV) leakage and the size of laser-induced lesions [13]. However, the precise mechanism by which ATN-161 ameliorates RNV remains unclear.

Apart from ischemia and hypoxia, inflammatory reactions also play an important role in RNV [2]. The NACHT, LRR, and PYD domains-containing protein 3 (NLRP3) (formerly known as cryopyrin, NALP3) is one member of the NOD-like receptor (NLR) protein family [14, 15]. Upon activation, NALP3 and adaptor protein apoptosis-associated speck-like protein containing a CARD (ASC) assemble a protein complex known as NALP3 inflammasome [16]. The assembled inflammasome triggers protease caspase-1, which then cleaves and produces proinflammatory cytokines interleukin-1β (IL-1β) and IL-18 from their inactive precursor forms [14]. Several reports have demonstrated that the activation of NLRP3 inflammasome is drawn into the inflammatory injury of retinal ischemia [17, 18]. These data suggest that NLRP3 inflammasome may play a key role in RNV.

In this study, we aimed to determine the effect and underlying mechanism after inhibiting integrin α5β1 by ATN-161 in Tet/opsin/VEGF transgenic mice, which highly express VEGF in photoreceptors, resulting in severe NV and significant leakage causing total exudative retinal detachment in 80 to 90% of mice after treatment with doxycycline [19, 20]. These studies may enable us to understand the molecular mechanisms of regulating RNV and lead to new potential pharmacotherapy in addition to anti-VEGF treatments for future antiangiogenic therapies.

## Materials and methods

### Reagents

ATN-161 (Qiangyao, St. Louis, MO and China). Primary antibody: integrin α5, NLRP3, ASC (Santa Cruz Biotechnology, Santa Cruz, CA, USA); cleaved-IL-1β (Millipore, Billerica, MA, USA); integrin β1, cleaved-caspase-1 (Abcam, Cambridge, MA, USA); β-actin (Cell Signaling Technology, Inc., Danvers, MA). Secondary antibodies (Cell Signaling Technology, Inc., Danvers, MA). Anti-mouse CD31(PECAM-1)-PE (eBioscience Inc., San Diego, CA); goat anti-rabbit immunoglobulin (IgG)-fluorescein isothiocyanate (FITC) (Santa Cruz Biotechnology). All cell culture reagents were bought from Invitrogen (Carlsbad, CA, USA).

### Animals

Tet/opsin/VEGF mice were kindly provided by Professor Peter Campochiaro (Johns Hopkins Hospital, Baltimore, MD) [19]. These double transgenic mice were completely normal until they were given a 2 mg/mL dosage of doxycycline in their drinking water to stimulate human VEGF_165_ expression in photoreceptors resulting in severe NV and significant leakage, leading to total exudative retinal detachment in 80–90% of mice within 5 days [19, 20]. The severe phenotype of retinal detachment has proven extremely useful to test efficacy of treatments [20]. In this study, all the mice used were specific pathogen free. Animal care and all procedures were carried out on the basis of the Health Guide for Care and Use of Laboratory Animals (National Institutes), the Scientific Investigation Board approval (SYXK-2003-0026, Shanghai Jiao Tong University School of Medicine, Shanghai, China).

### Quantitative reverse transcription polymerase chain reaction (RT-PCR)

RNA was extracted from retinas and human retinal endothelial cells (HRECs) samples in accordance with the manufacturer’s protocols. Assessing RNA quality and quantity, we chose 2 μg of RNA to reverse transcribe, and then obtained complementary DNA (cDNA). The cDNA was used for quantitative RT-PCR with the iQ SYBR Green mix (Roche, Basel, Switzerland) on an ABI 7500 Real-time PCR system (Applied Biosystems, CA, USA). Primers used included: mouse-integrin α5 (forward: 5′-CGTTGAGTCATTCGCCTCTGG-3′, reverse: 5′- GTGCCCGCTCTTCCCTGTC-3′) [21], mouse-integrin β1 (forward: 5′-TGGAAAATTCTGCGAGTGTG-3′, reverse: 5′-GCATTCACAAACACGACACC-3′), mouse-NLRP3 (forward: 5′-TCCTGGTGACTTTGTATATGCGT-3′, reverse: 5′-TTCTCGGGCGGGTAATCTTC-3′), mouse-ASC (forward: 5′-GCTGAGCAGCTGCAAACGA-3′, reverse: 5′-ACTTCTGTGACCCTGGCAATGA-3′) [22], mouse-caspase-1 (forward: 5′-TGCCTGGTCTTGTGACTTGGA-3′, reverse: 5′- CCTATCAGCAGTGGGCATCTGTA -3′) [22], mouse-IL-1β (forward: 5′-TGCCACCTTTTGACAGTGATG-3′, reverse: 5′-AAGGTCCACGGGAAAGACAC-3′) [23], mouse-cyclophilin A (forward: 5′-CAGACGCCACTGTCGCTTT-3′, reverse: 5′-TGTCTTTGGAACTTTGTCTGCAA-3′) [23], human-NLRP3 (forward: 5′-CTTCAGGTGTTGGAATTAGAC-3′, reverse: 5′-GCACTTCACAGAACATCAT-3′) [24], human-ASC (forward: 5′-TTGGACCTCACCGACAAGC-3′, reverse: 5′-TATAAAGTGCAGGCCCTGGTG-3′), human-caspase-1 (forward: 5′-GGACAAGTCAAGCCGCACA-3′, reverse: 5′- CATGTCCGAAGCAGTGAGAT-3′) [25], human-IL-1β (forward: 5′-TGCCACCTTTTGACAGTGATG-3′, reverse: 5′-CCTATCAGCAGTGGGCATCTGTA-3′) [24], and human-GAPDH (forward: 5′-GGGAAACTGTGGCGTGAT-3′, reverse: 5′-GAGTGGGTGTCGCTGTTGA-3′).

### Western blot analysis

Proteins from retinas and cell lysate were loaded onto 8% (or 12%) sodium dodecyl sulfate (SDS) polyacrylamide gel electrophoresis, and then transferred to polyvinylidinedifluoride membranes (Millipore). These blots were incubated with primary antibodies against integrin α5, integrin β1, NLRP3, ASC, cleaved-caspase-1, cleaved-IL-1β, and β-actin overnight at 4 °C after 5% non-fat milk. Next, these blots were incubated with horseradish peroxidase (HRP)-conjugated secondary antibodies for 1 h. An enhanced chemiluminescence kit (ECL, Millipore) was used to visualize these immunoreactive bands.

### Frozen section analysis

The eyes from Tet/opsin/VEGF transgenic mice were harvested carefully (avoiding artificial retinal detachment) after 5 days treated with doxycycline, frozen in the Tissue-Tek OCT media, prepared into 10-μm-thick slices from the cornea to the optic nerve. The images were photographed with an optical microscope. The retinal detachment state of the slices around the optic nerve was examined for total retinal detachment (TRD), partial retinal detachment (PRD), or no retinal detachment (no RD) [26].

### Immunofluorescence staining

The eyes from adult Tet/opsin/VEGF transgenic mice were prepared into 10-μm-thick slices. These sections were fixed with 4% paraformaldehyde at room temperature, and then rinsed in phosphate buffered saline (PBS). After incubation in 0.5% TritonX-100, the sections were incubated in 5% bovine serum albumin, incubated with polyclonal rabbit anti-mouse-integrin α5, integrin β1, NLRP3, or ASC antibody (Santa Cruz Biotechnology), and then incubated in the mixture of FITC anti-rabbit IgG antibody (Cell Signaling Technology) and anti-mouse CD31(PECAM-1)-PE (eBioscience). After 5 min of incubation in 4′, 6-diamidino-2-phenylindole (DAPI), the sections were photographed with a fluorescence microscope. The HRECs were cultured on the cell culture slide (Millipore) and treated with 20 ng/mL VEGF alone, or in combination with 10 μM ATN-161 for 24 h. After washing with PBS, the HRECs were fixed in 4% paraformaldehyde, and then permeabilized in 0.5% Triton X-100. Subsequently, the HRECs were blocked in 5% bovine serum albumin and incubated in anti-NLRP3 or ASC antibody (Santa Cruz), followed by incubation in the FITC anti-rabbit IgG antibody (Invitrogen). After incubation of 5 min in DAPI, the cell samples were examined under a fluorescence microscope and the images were acquired.

### Cell culture and stimulation

The culture conditions of HRECs (ScienCell) included an atmosphere (containing 5% CO_2_, humidified, 37 °C), endothelial cell medium (supplemented with 100 U/mL penicillin-streptomycin, 5% fetal bovine serum) [2]. The cells were incubated in media with 20 ng/mL VEGF with or without 10 μM ATN-161 for 12 h to detect NLRP3, ASC, caspase-1, IL-1β mRNA expression by RT-PCR and for 24 h to detect protein expression by western blot assay and immunofluorescence staining.

### Statistical analysis

Classification data analysis used the chi-square test. Multiple comparisons used Student-Newman-Keuls method. The differences between two groups adopted the two-tailed Student’s *t* test. All the statistical analyses were shown using SAS 9.0 software (SAS Institute Inc., Cary, NC, USA). All data are performed as mean values ± standard error of mean (SEM). A value of *P* < 0.05 was deemed to be statistically significant.

## Results

### Integrin α5β1 expression was increased in adult Tet/opsin/VEGF transgenic mice treated with doxycycline

**Fig. 1:**
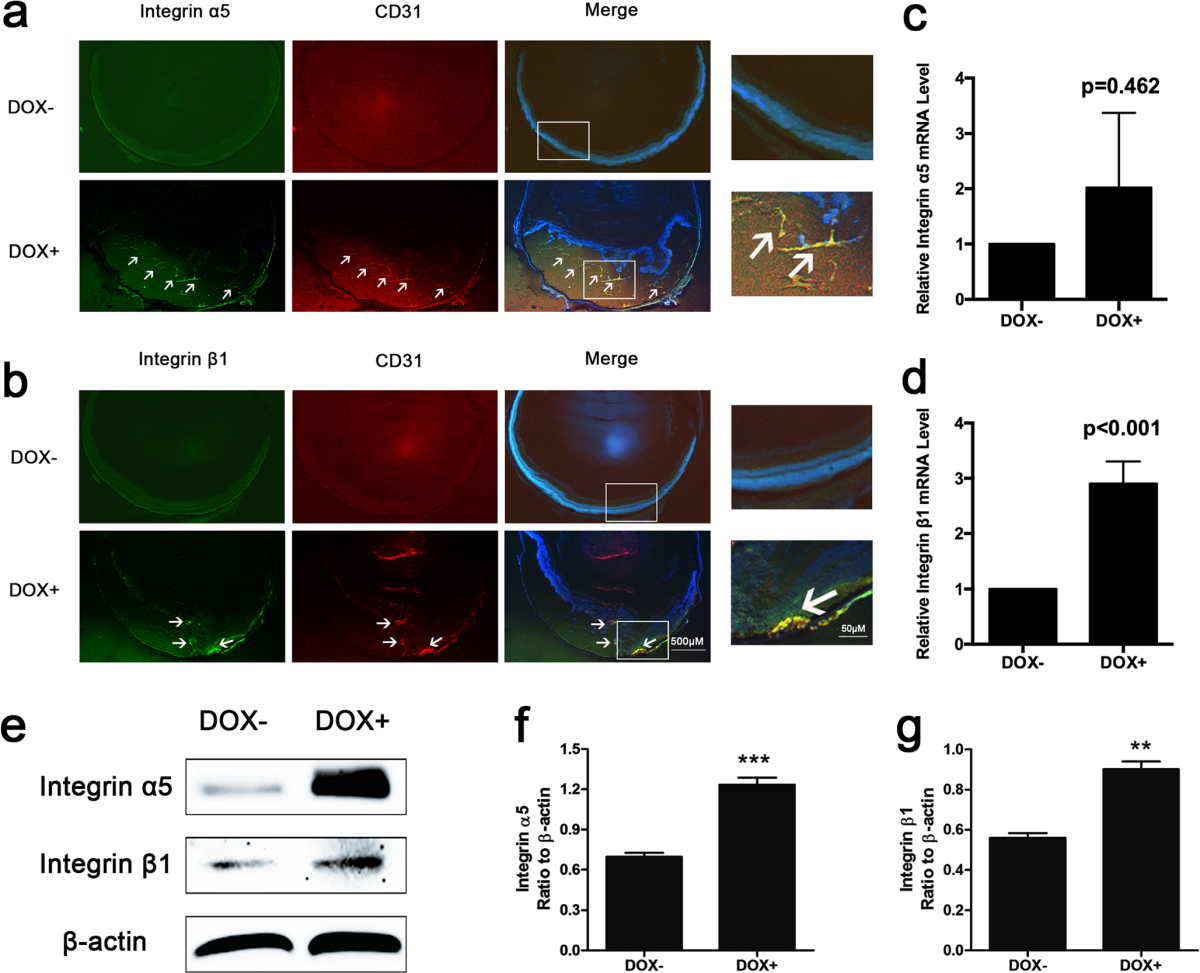
Expression levels of integrin α5β1 examined by immunofluorescent staining, RT-PCR, and western blot assays in retinas from Tet/opsin/VEGF transgenic mice. Eyes were collected after 5 days of treatment with doxycycline (DOX+) or normal treatment (DOX−). The frozen sections were immunofluorescently stained with integrin α5 (**a**) or integrin β1 (**b**) and CD31 (a marker for VEC). Each positive area was marked with arrowhead. Total RNA of retinas was isolated and mRNA expression of integrin α5 (**c**, *n* = 8) and integrin β1(**d**, n = 8) was determined. 2^-ΔΔCT^ method was used to calculate the relative fold change. Western blot assays were conducted to evaluate the protein expression of integrin α5β1 in DOX− and DOX+ mice (**e**, *n* = 3). Relative protein expression levels of integrin α5 and integrin β1 normalized to β-actin are shown in **f** and **g**. All data are shown as mean ± SEM from three independent experiments. Student *t* test were used to carry out statistical analysis

After 5 days of treatment with doxycycline, mRNA expression of integrin α5 was not elevated significantly (Fig. 1c, *P* = 0.462) whereas protein expression was increased significantly (Fig. 1f, *P* < 0.001). Both mRNA and protein expression of integrin β1 were elevated significantly compared with controls (Fig. 1d, *P* < 0.001; Fig. 1g, *P* = 0.002). The results of immunofluorescence staining showed that integrin α5 and β1 expression levels in Tet/opsin/VEGF transgenic mice with doxycycline treatment were even higher, and the merged images showed that integrin α5 and integrin β1were mostly localized in vascular endothelial cells (VECs) (Fig. 1a, b). These findings indicate that integrin α5β1 may be mainly derived from RNV endothelial cells and play a significant role in the retinal detachment of Tet/opsin/VEGF mice.

### ATN-161 inhibited integrin α5β1 expression in adult Tet/opsin/VEGF transgenic mice treated with doxycycline

**Fig. 2:**
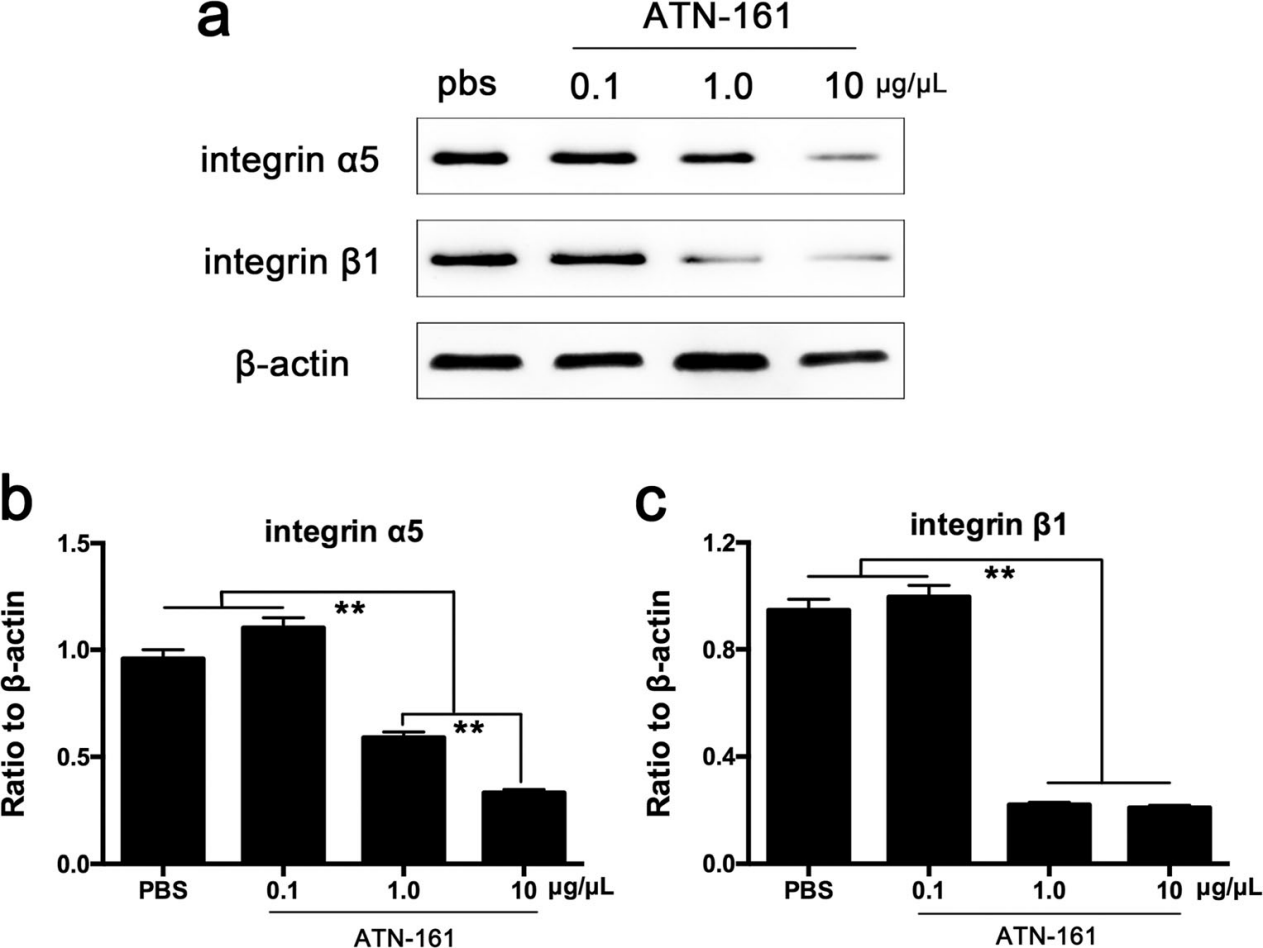
Influence of ATN-161 on integrin α5β1 expression in retinas from adult Tet/opsin/VEGF transgenic mice with doxycycline treatment. Adult mice were intravitreously injected with 1 μL of ATN-161 in one eye with different concentrations (0.1, 1, and 10 μg/μL) and PBS in the other one. **a** Integrin α5 and integrin β1 expression levels were detected by western blot assay (*n* = 3). **b**, **c** Quantification of the intensity of target protein bands normalized relative to β-actin. Three independent experiments were conducted for statistical analysis (mean ± SEM), and Student-Newman-Keuls (SNK) was used to perform statistical analysis for multiple comparisons. (**P < 0.01)

The intravitreal injection of ATN-161 followed the designed concentration gradient (0, 0.1, 1, and 10 μg/μL) to determine the lowest effective dosage in adult Tet/opsin/VEGF transgenic mice with doxycycline treatment. The analysis of western blot results revealed that ATN-161 significantly reduced the expression of integrin α5β1 at concentrations of 1 and 10 μg/μL, and this seemed to present a dose-independent manner (Fig. 2). Therefore, we chose 1 μg/μL to conduct subsequent experiments.

### Inhibiting integrin α5β1 by ATN-161 ameliorated retinal detachment of Tet/opsin/VEGF transgenic mice

**Fig. 3:**
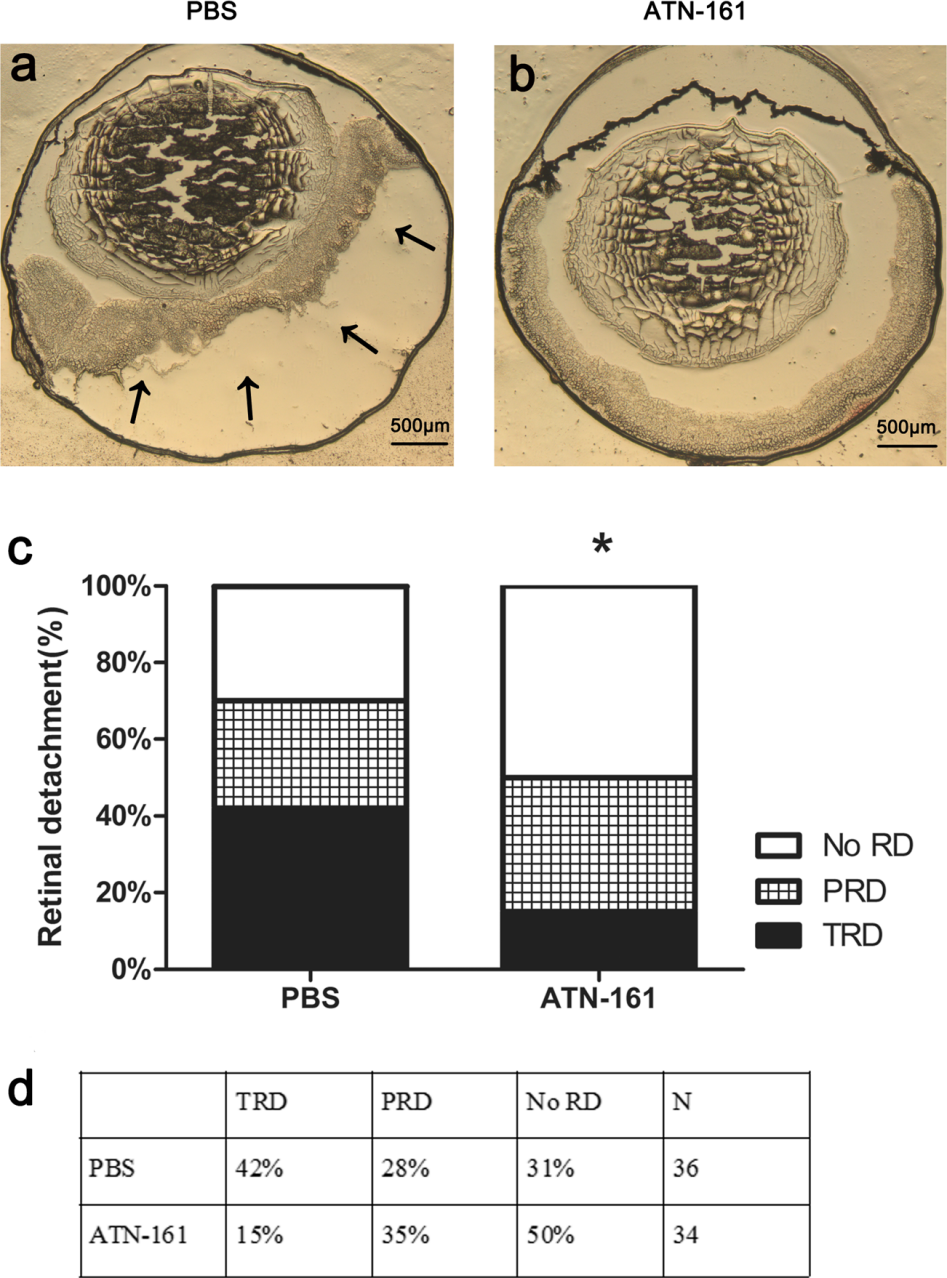
Influence of ATN-161 on retinal detachment in adult Tet/opsin/VEGF transgenic mice treated with doxycycline. Tet/opsin/VEGF mice were intravitreously injected with PBS (1 μL, **a**, *n* = 36) in one eye and ATN-161(1 μL of 1 μg/μL, **b**, *n* = 34) in the other eye. After 5 days of doxycycline treatment of drinking water, the state of retinal detachment was examined under an optical microscope for total retinal detachment (TRD), partial retinal detachment (PRD), or no retinal detachment (no RD). Chi-square test was used to analyze data (**c**, mean ± SEM), *P < 0.05. The table in **d** showed the percentage of various retinal detachment types

Tet/opsin/VEGF transgenic mice received an intravitreous injection of ATN-161(1 μL of 1 μg/μL) in one eye and PBS (1 μL) in the other eye after administration of intraperitoneal anesthesia. Compared with the control eyes, there was significantly ameliorated retinal detachment in ATN-161 injected eyes (Fig. 3, *P* < 0.05). These data imply that integrin α5β1 plays a supportive part in retinal detachment in Tet/opsin/VEGF mice.

### NLRP3 inflammasome expression increased in adult Tet/opsin/VEGF transgenic mice with doxycycline treatment

**Fig. 4:**
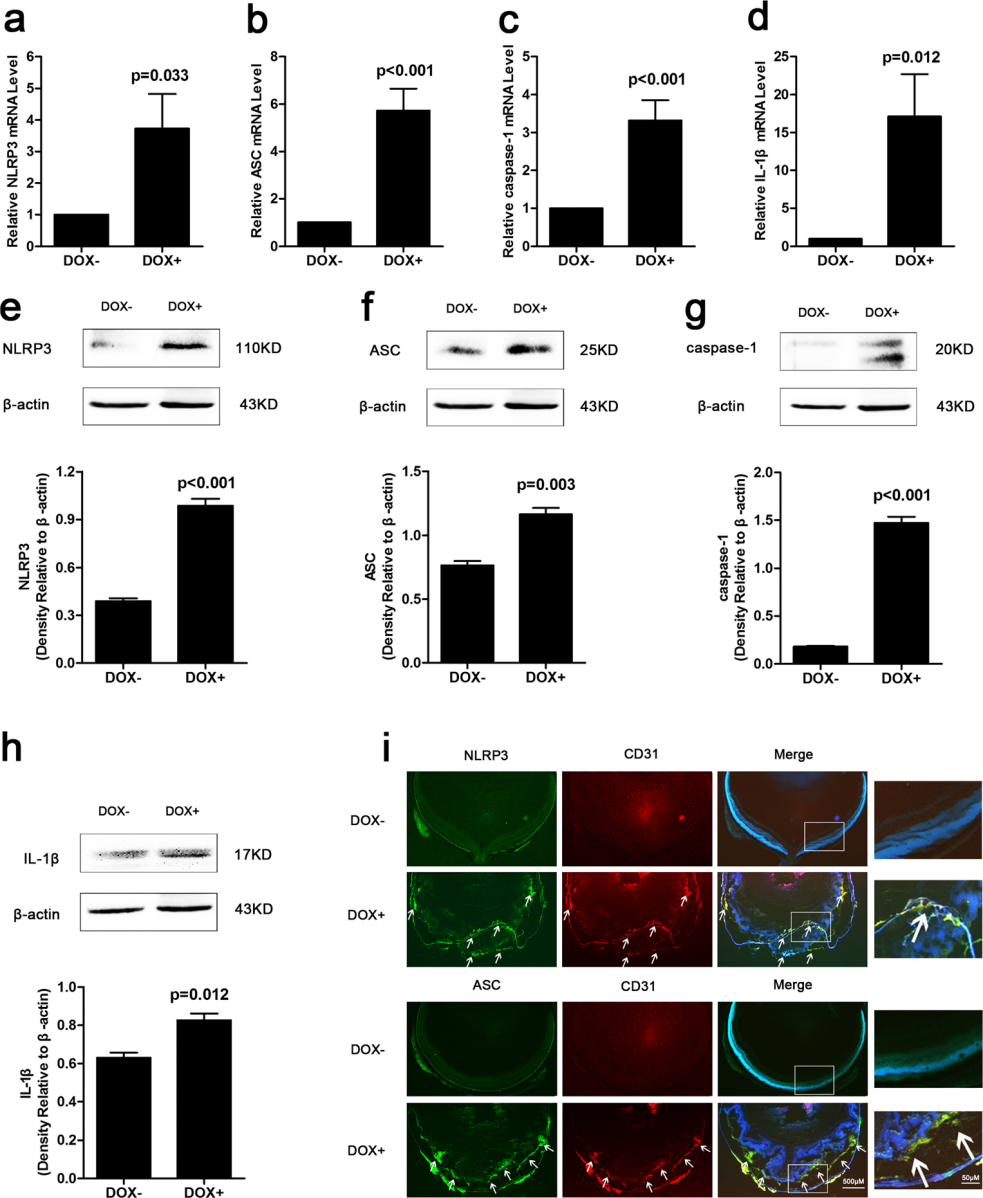
NLRP3 inflammasome expression levels were detected in retinas from Tet/opsin/VEGF transgenic mice. Total RNA of retinas was isolated and the mRNA expression levels of NLRP3 (**a**, *n* = 6), ASC (**b**, *n* = 8), caspase-1 (**c**, *n* = 8), and IL-1β (**d**, *n* = 8) were detected by real-time RT-PCR in DOX− and DOX+ mice. 2^-ΔΔCT^ method was used to count up the relative fold change. Protein expression levels of NLRP3 (**e**, *n* = 3), ASC (**f**, *n* = 3), cleaved caspase-1 (**g**, *n* = 3) and cleaved IL-1β (**h**, *n* = 3) were determined using western blot assays. All data are shown as mean ± SEM from three independent experiments. Student’s *t* test was used to carry out statistical analysis. Immunofluorescent staining of ocular frozen sections was used to test expression of NLRP3, ASC, and CD31 (**i**). Each positive area was marked with arrowhead

Our findings demonstrated that VEGF promoted the mRNA upregulation of NLRP3 (Fig. 4a, *P* = 0.033), ASC (Fig. 4b, *P* < 0.001), caspase-1 (Fig. 4c, *P* < 0.001), and IL-1β (Fig. 4d, *P* = 0.012) in adult Tet/opsin/VEGF transgenic mice treated with doxycycline. The protein expression levels of NLRP3 (Fig. 4e, *P* < 0.001), ASC (Fig. 4f, *P* = 0.003), cleaved-caspase-1(Fig. 4g, *P* < 0.001), and mature IL-1β (Fig. 4h, *P* = 0.012) were also increased. Immunofluorescence staining also showed that NLRP3 and ASC (Fig. 4i) significantly increased and co-localization with RNV endothelial cells occurred in adult Tet/opsin/VEGF transgenic mice treated with doxycycline. These results demonstrate the stimulatory effect of VEGF on NLRP3 inflammasome activation in RNV endothelial cells of Tet/opsin/VEGF transgenic mice with doxycycline treatment.

### ATN-161 reduced NLRP3 inflammasome activation in adult Tet/opsin/VEGF transgenic mice

**Fig. 5:**
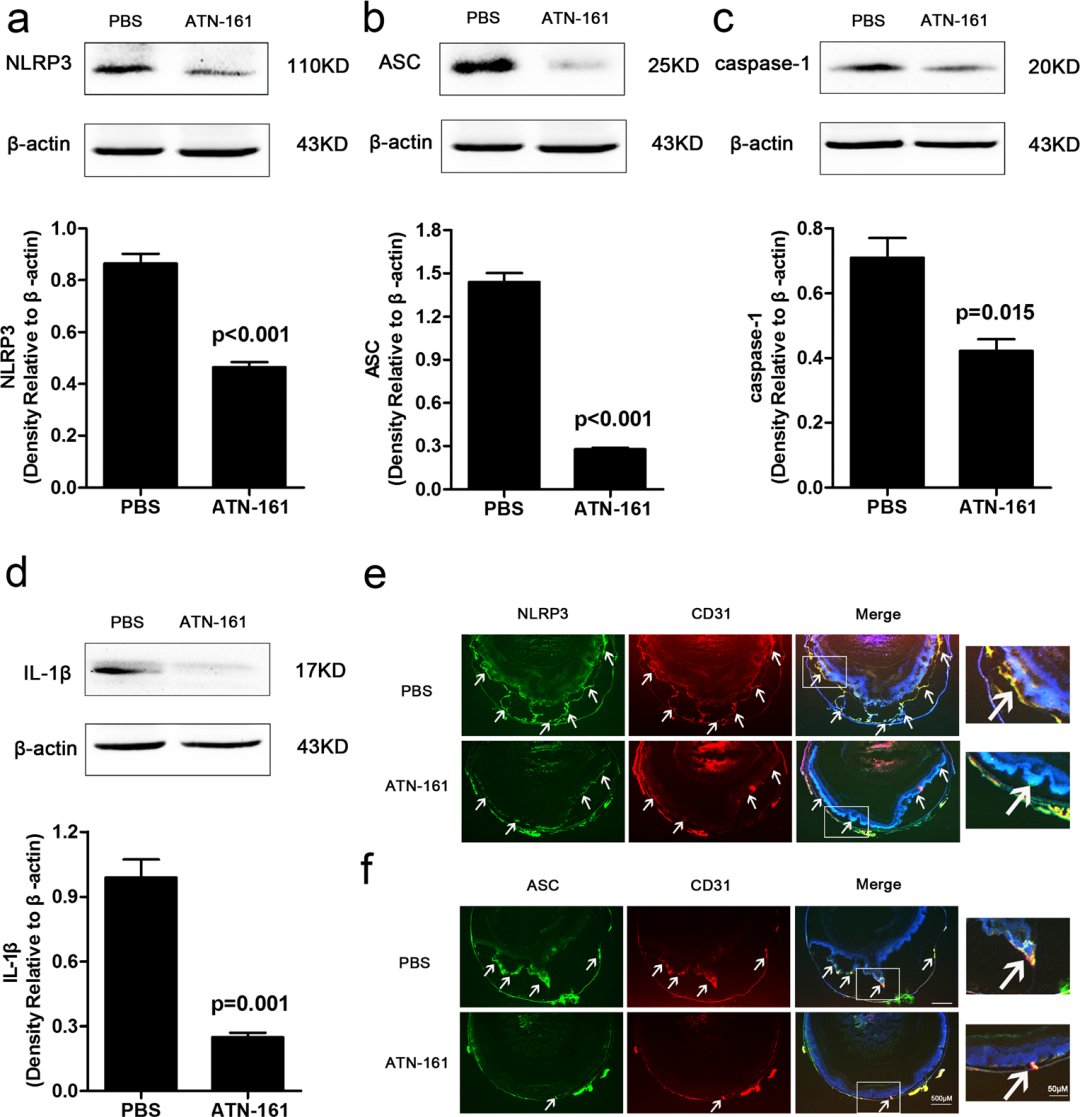
Effect of ATN-161 on NLRP3 inflammasome activation in retinas from Tet/opsin/VEGF transgenic mice with doxycycline treatment. The image showed that retinal protein expression of NLRP3 (**a**, *n* = 3), ASC (**b**, *n* = 3), cleaved caspase-1 (**c**, *n* = 3), and cleaved IL-1β (**d**, *n* = 3) from mice after intervention with ATN-161 or PBS. Student’s *t* test was used to analyze data. The values represented mean ± SEM from independent experiments (three times). The expression of NLRP3 (**e**), ASC (**f**), and CD31 in ocular frozen section was tested by immunofluorescent staining. The arrowheads showed positive results for each labeling staining

The eyes from transgenic mice treated with ATN-161 or PBS were collected for western blot and immunofluorescence staining assays at proper time. The results showed that ATN-161 significantly inhibited the protein expression of NLRP3 (Fig. 5a, *P* < 0.001), ASC (Fig. 5b, *P* < 0.001), cleaved caspase-1 (Fig. 5c, *P* = 0.015), and mature IL-1β (Fig. 5d, *P* = 0.001). These findings suggest that VEGF might stimulate the activation of NLRP3 inflammasome through integrin α5β1, and then promote RNV in Tet/opsin/VEGF transgenic mice. These data suggest that inhibition of integrin α5β1 ameliorated VEGF-induced retinal detachment by suppressing the activation of NLRP3 inflammasome in Tet/opsin/VEGF transgenic mice.

### ATN-161 reduced NLRP3 inflammasome activation in HRECs treated with VEGF

**Fig. 6:**
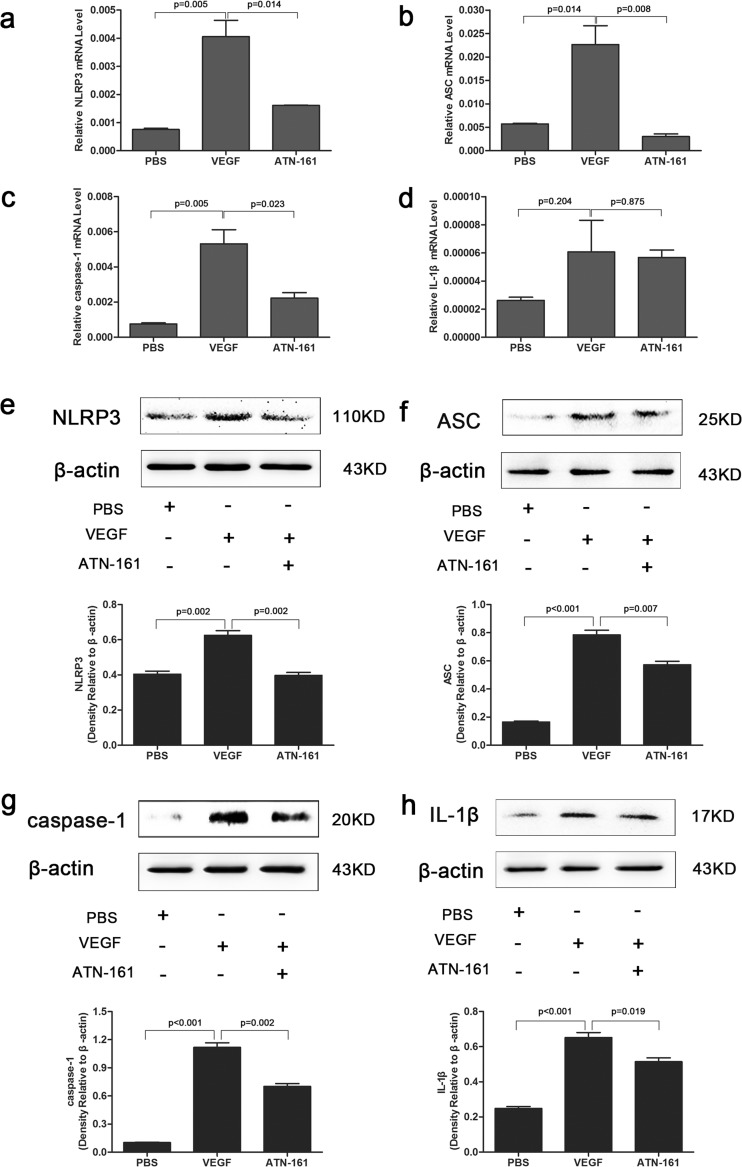
Impact of ATN-161 on NLRP3 inflammasome expression tested by RT-PCR and western blot assay in VEGF-treated HRECs. **a**–**d** Real-time RT-PCR detected NLRP3, ASC, caspase-1, and IL-1β expression levels in HRECs with 20 ng/mL VEGF alone or in association with 10 μM ATN-161. 2^-ΔCT^ method was used to count up the relative fold change. **e**–**h** Western blot assay analyzed NLRP3, ASC, cleaved-caspase-1, and cleaved-IL-1β expression levels in HRECs treated with VEGF alone or in combination with ATN-161. Student’s *t* test was used to analyze data. The values represented mean ± SEM from independent experiments (three times)

**Fig. 7:**
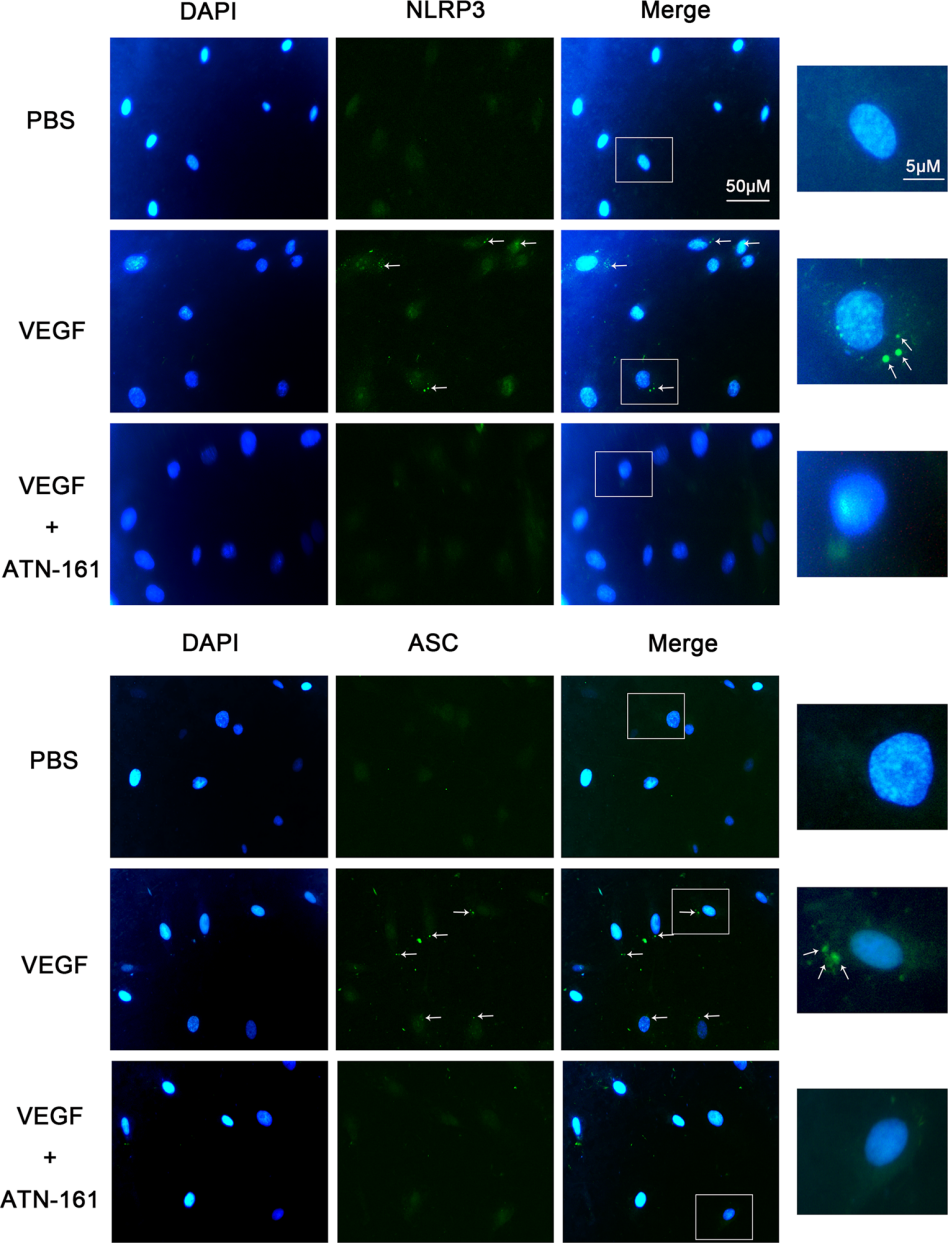
Immunofluorescent staining for NLRP3 and ASC in HRECs. Immunofluorescent staining assay analyzed NLRP3 (green) and ASC (green) expression in HRECs treated with 20 ng/mL VEGF alone or in association with 10 μM ATN-161. Each positive area was marked with arrowhead. The repeated experiments (three times) obtained similar results. The pictures displayed representative results of each group

In this study, we found a significant increase in NLRP3, ASC, cleaved caspase-1and mature IL-1β mRNA (Fig. 6a–d) and protein expression (Fig. 6e–h) in the HRECs treated with VEGF as compared to PBS groups, and this upregulation was dramatically inhibited in the ATN-161 group. Immunofluorescence staining assays (Fig. 7) showed more staining for NLRP3 and ASC in VEGF groups compared with PBS groups, and faint staining after treatment with ATN-161. The merged images demonstrate that NLRP3 and ASC were located in the cytoplasm of HREC indicating that retinal endothelial cells might be the main source of NLRP3 and ASC secretion. These data demonstrate that VEGF can promote the secretion of NLRP3 inflammasome and inhibiting the expression of integrin α5β1 by ATN-161 can decrease the NLRP3 inflammasome secretion induced by VEGF.

## Discussion

ROP and DR [2] are common causes of vision loss associated with RNV. Currently, VEGF is known to play a significant part in neovascular diseases and anti-VEGF therapy is beneficial in patients. However, failure of anti-VEGF therapy in some patients [27] reminds us that the molecular mechanisms of neovascular diseases need to be further explored. Integrins consist of alpha and beta subunits and form heterodimeric glycoprotein receptors through a variety of combinations mediating cell-cell or cell-extracellular matrix interactions [28]. Integrins binding to ligands play important roles in normal cellular functions including cell proliferation, migration, and differentiation, as well as in the pathogenesis of chronic inflammation [29]. In our study, we evaluated the effect of inhibiting integrin α5β1 expression on retinal detachment in adult Tet/opsin/VEGF mice and investigated the underlying mechanisms in vitro and in vivo. Integrin α5β1 is one member of integrin family as well as a specific receptor of fibronectin [9]. Some tumor studies showed that integrin α5β1 is upregulated in tumor angiogenesis, but not in normal vasculature [11]. We detected the expression levels of integrin α5β1 in Tet/opsin/VEGF mice and found an increased expression in adult mice treated with doxycycline. This was consistent with existing research results that integrin α5β1 and fibronectin were upregulated in growth factor-induced NV [11], whereas expression of integrin α5β1 was low in quiescent vascular cells [11, 12] and indicated integrin α5β1may play an important role in retinal detachment progression. We also explored the positional relationship between integrin α5β1 and NV in detached retinas. Immunofluorescence staining showed integrin α5β1 located in the retina near the choroid and displayed co-localization with RNV. These results showed RNV endothelial cells expressed higher integrin α5β1, implying a close relationship between integrin α5β1 and RNV. To further explore the role of integrin α5β1 in RNV, we adopted the method of inhibiting integrin α5β1 expression by ATN-161 and analyzing the effect of integrin α5β1 inhibition on massive RNV leakage-induced retinal detachment. Retinal detachment was significantly ameliorated in adult Tet/opsin/VEGF mice with integrin α5β1 blockade. This was in accordance with prior research, which showed that integrin α5β1 inhibition by ATN-161 significantly decreased CNV leakage, NV, and the size of laser-induced lesions [13]. However, the precise mechanisms by which ATN-161 ameliorated RNV remain unclear.

We wondered how integrin α5β1 participated in massive RNV leakage-induced retinal detachment process and explored the possible mechanisms. As we know, inflammation is essential for host defense against infections, but the chronic or excessive production of IL-1β is harmful to the individual [16, 25, 29]. Thus, targeting IL-1β or IL-1β receptor was used as a potential therapeutic approach for inflammatory diseases by targeting the NLRP3 activation pathway [30, 31]. Several reports have demonstrated that inflammation participated in the pathogenesis of ocular NV [32, 33] and the NLRP3 inflammasome activation was involved in the inflammatory injury of retinal ischemia [17, 18]. Some findings indicated that inhibiting NLRP3 ameliorated ischemic injury in animal and cellular models [34]. The central role of activation of NLRP3 inflammasome in retinal pigment epithelium cells as well as in models of age-related macular degeneration has also been reported [35]. The activation of caspase-1 and increased expression of IL-1β were well documented in DR with an important proinflammatory role in mediating microvascular degeneration and retinal endothelial cell dysfunction [35]. In our experiments, the mRNA and protein expression levels of NLRP3, ASC, caspase-1, and IL-1β were significantly upregulated in adult Tet/opsin/VEGF mice with doxycycline treatment. Immunostaining showed co-localization of NLRP3 (or ASC) with RNV endothelial cells, and all of them displayed more staining compared with controls. All these data suggested that NLRP3 inflammasome participated in the RNV and retinal detachment process in Tet/opsin/VEGF mice. Some research showed the invasion-integrin interaction provided the first signal for NLRP3 inflammasome activation in intestinal epithelial cells [36]. A recent report also demonstrated that *Treponema denticola* surface protein Td92 bound to α5β1 integrins, leading to the full NLRP3 inflammasome activation in THP-1 monocytes [29]. To explore the influence of ATN-161 on NLRP3 inflammasome in vivo, the protein expression levels of NLRP3, ASC, cleaved caspase-1, and mature IL-1β were detected in the retinas from adult Tet/opsin/VEGF mice that received ATN-161 or PBS. We found a significant decrease in NLRP3, ASC, cleaved caspase-1 and mature IL-1β after inhibiting integrin α5β1 by ATN-161 in Tet/opsin/VEGF mice. The results suggested that inhibition of integrin α5β1 could reduce the NLRP3 inflammasome activation in adult Tet/opsin/VEGF mice with doxycycline treatment.

In an in vitro assay, we observed similar results, namely, a significantly increased mRNA and protein expression of NLRP3, ASC, cleavage of caspase-1, and mature IL-1β in the HRECs treated with VEGF as compared to PBS groups, and this upregulation was dramatically inhibited in the ATN-161 group. Immunofluorescence staining assays showed more staining for NLRP3 and ASC in VEGF groups and faint staining after treatment with ATN-161. The merged pictures demonstrated that NLRP3 and ASC were located in the cytoplasm of HREC, indicating that retinal endothelial cells might be the main source of NLRP3 and ASC secretion. All these data clarified that VEGF could promote the secretion of NLRP3 inflammasome; this phenomenon was reversed after inhibiting the expression of integrin α5β1 by ATN-161. However, the limitations of this study include the effects of targeting integrin α5β1 combined with anti-VEGF agents, and the precise molecular mechanisms by which NLRP3 inflammasome regulated RNV remain to be illuminated by further study.

In summary, our study elucidated that integrin α5β1 participated in significant RNV leakage-induced retinal detachment in adult Tet/opsin/VEGF mice treated with doxycycline for the first time. Intravitreous injection of ATN-161 inhibiting integrin α5β1 stifled VEGF-induced retinal detachment. The probable mechanisms include integrin α5β1 stimulated NLRP3 inflammasome expression and mature IL-1β secretion, and then resulted in severe NV and significant leakage that caused total exudative retinal detachment.

## Conclusion

Our data indicate that inhibition of integrin α5β1 ameliorates VEGF-induced retinal detachment by suppressing NLRP3 inflammasome signaling in Tet/opsin/VEGF transgenic mice, which could be beneficial for the treatment of RNV diseases.
